# Neither increasing the frequency of sow feedings nor decreasing the interval between feedings prior to farrowing reduced piglet stillbirths

**DOI:** 10.1093/tas/txae150

**Published:** 2024-10-23

**Authors:** Danielle C Johnson, Jeremy G Perez, Jorge Estrada, Deanne Corzatt, Michael W Welch, Eric Parr, Dustin D Boler

**Affiliations:** Carthage Innovative Swine Solutions, Carthage Veterinary Service Ltd., Carthage, IL 62321, USA; Carthage Innovative Swine Solutions, Carthage Veterinary Service Ltd., Carthage, IL 62321, USA; Carthage Innovative Swine Solutions, Carthage Veterinary Service Ltd., Carthage, IL 62321, USA; Carthage Innovative Swine Solutions, Carthage Veterinary Service Ltd., Carthage, IL 62321, USA; Carthage Innovative Swine Solutions, Carthage Veterinary Service Ltd., Carthage, IL 62321, USA; Carthage Innovative Swine Solutions, Carthage Veterinary Service Ltd., Carthage, IL 62321, USA; Carthage Innovative Swine Solutions, Carthage Veterinary Service Ltd., Carthage, IL 62321, USA

**Keywords:** farrowing, feeding frequency, gestation, meal interval, stillbirth, pig

## Abstract

Farrowing durations that exceed 240 min cause stillborn rates to increase. Therefore, feeding strategies in late gestation have been studied to mitigate the negative consequences of extended farrowing durations. A total of 1,501 sows (PIC 1050 Camborough) were used for this study at two individual farms near Carthage, IL. Farm 1 (758 sows) was a porcine reproductive and respiratory virus (PRRSv) stable (previously experienced a PRRSv outbreak and is currently vaccinated for PRRSv) with an older parity structure (3.67). Farm 2 (743 sows) was PRRSv positive (sows with pigs demonstrating Ct values <36 determined by pig processing fluids) and had a younger parity structure (2.96). Sows were moved into farrowing rooms at approximately day 112 of gestation and started on their respective treatment. Treatment 1 sows were fed 1 meal of 2.27 kg per day at 0600 h. Treatment 2 sows were fed two equal meals of 1.13 kg (2.27 kg total) at 0600 and 1400 h. Treatment 3 sows were fed two equal meals of 1.13 kg (2.27 kg total) at 0600 and 1800 h. Treatment 4 sows were fed three equal meals of 0.77 kg (2.27 kg total) at 0600, 1400, and 2200 h. Treatments were assigned to farrowing rooms in a replicated 4 × 4 Latin Square arrangement of treatments where each treatment was fed in each room one time at each farm. Daily feed intakes were recorded from the time sows were introduced to the farrowing room until 5 d after farrowing. The total number of pigs born, pigs born alive, stillbirths, and mummies were recorded for each litter within 24 h of farrowing. Live pigs were weighed as a group to record litter birth weight. Pig mortality and morbidities were recorded until 5 d after farrowing. The total number of pigs born and pigs born alive were not different (*P* ≥ 0.59) among treatments. The number of stillborn pigs was not different (*P* = 0.33) among treatment 1(1.15 ± 1.41), treatment 2 (1.20 ± 1.36), treatment 3 (1.30 ± 1.46), and treatment 4 (1.14 ± 1.28). Sows fed three times per day at 8 h intervals tended (*P* = 0.08) to reduce the percentage of sows farrowed under supervision compared with sows fed once a day. Sows fed twice per day at 12 h intervals reduced (*P* = 0.01) the percentage of sows provided assistance compared with feeding sows once per day. Feeding a sow one meal of 2.27 kg, two meals (2.27 kg total), or three meals (2.27 kg total) of feed a day before farrowing did not reduce the number of stillborn piglets regardless of farm health status or parity structure.

## Teaser

The total number of piglets born per sow continues to increase year over year and is important to a farm’s profitability. However, farrowing duration has also increased and is connected to a greater number of stillborn piglets per litter. Feeding strategies such as changing the frequency and amount of feed a sow is allocated prior to farrowing have been of focus to mitigate the number of stillborn piglets. Sows were fed four different meal frequency strategies at two farms with different health statuses and parity structures. Increasing the frequency of feedings before farrowing did not reduce the number of stillborn piglets at either farm.

## INTRODUCTION

The total number of pigs born in each litter has increased to three more piglets per litter today compared to 20 years ago (USDA-NASS, 2023) and that rate of increase is projected to continue. Increasing the number of piglets born per litter is an important economic trait and often a breeding goal of pig producers. A major concern with increasing the total number of pigs born is that farrowing duration also increases (Oliviero et al., 2010; Tucker et al., 2022).

Farrowing durations that exceed 240 min have an increase in stillborn rate compared to farrowing durations under 240 min (Ju et al., 2021). The average number of total pigs born per litter is 15.3 (MetaFarms, 2023). This means that the birthing interval must be approximately 15 min to maintain a 240-min farrowing duration and minimize stillbirths, but this is often not achieved. It is thought sows deplete energy reserves during extended farrowing events resulting in an increase of stillbirths (Oliviero et al., 2010; Feyera et al., 2018).

Therefore, feeding strategies in late gestation have become a focus area to provide energy needed to aid sows with extended farrowing durations (Miller and Kellner, 2020; Oliveria et al., 2020; Tucker et al., 2022). Most commercial farms determine feeding frequency based on farm logistics and available resources. Farms that individually feed sows may be limited to feeding during staffed hours and become dependent on labor availability. Farms with automatic feeding systems may have more flexibility with the time and frequency of feeding prior to farrowing.

Results from increasing the frequency of feedings prior to farrowing demonstrated inconsistent results. Miller and Kellner (2020) reported that farrowing duration and still birth rate decreases as meal frequency increased from one to two meals a day prior to farrowing. However, daily feed allocations were changed from 1.8 to 5.4 kg in addition to increasing from one to two meals a day. Miller and Kellner (2020) did not report the same benefits from increasing frequency to two meals a day when feeding sows 5.4 kg of feed a day. Tucker et al. (2022) reported the benefits of increasing feeding frequency in sows with farrowing durations under 3.47 h. There were no differences in the number of pigs stillborn when combining sows with farrowing durations above and below 3.47 h. Gourley et al. (2020) reported no differences in farrowing duration and stillborn rate with an increase in feed frequency prior to farrowing. It is likely differences in nutrient density and total feed allocated during each feeding contributed to the discrepancy in results among published literature. Another factor that may have contributed to the inconsistent results is assistance protocols. Assistance protocols with longer periods of time before intervening could influence farrowing duration and stillborn rate. Tucker et al. (2022) only provided assistance after a sow showed distress or 45 min had passed since the last piglet was born. Gourley et al. (2020) assisted after 30 min of no farrowing progress. It is unclear what assistance protocol Miller and Kellner (2020) used. Therefore, this study aimed to test four different feeding strategies by altering frequency before farrowing on 1,501 total sows at two different commercial sow farms in Western Illinois. The hypothesis was that stillborn pigs per litter would decrease as the interval between a sow’s last meal and farrowing also decreased, even when overall feed allocation is maintained.

## MATERIALS AND METHODS

All experimental procedures were reviewed and approved by the Carthage Veterinary Services IACUC committee (protocol #2023-17).

### Animals and Housing

A total of 1,501 sows (PIC 1050 Camborough) were used for this study at two separate farms near Carthage, Illinois. Farm 1 was porcine reproductive and respiratory virus (PRRSv) stable (previous PRRSv outbreak and currently vaccinated for PRRSv) with an older parity structure (3.67). Farm 2 was PRRSv positive (sows with pigs demonstrating Ct values < 36 determined by pig processing fluids) and had a younger parity structure (2.96). Sows were moved from gestation pens into farrowing rooms at a target of 112 d of gestation. Upon entering the farrowing room, sows were weighed, measured with a caliper at the last rib (scale 1–20); <8 considered thin, >11 considered over-conditioned (Knauer and Baitinger, 2015), and visually assigned a body condition score (BCS). Each farrowing stall (1.5 m × 2.1 m total, with 0.6 m × 2.1 m for sows) had a nipple waterer and feeder for the sow and a heat lamp for the pigs.

### Diets, Feeding, and Experimental Design

All sows received the same standard lactation diet (Table 1), which was formulated to meet or exceed the current National Research Council’s (2012) guidelines (Table 2) and PIC’s recommendations for lactating sows. Diets were manufactured at a commercial feed mill in Carthage, IL. Sows were fed on the schedule that corresponded with treatment from the time they were moved into the farrowing room until farrowing occurred. After farrowing, they were allowed ad libitum access to feed. Feed was added to the feeder, and feed refusals were recorded to calculate the average daily intake. Daily feed intake was recorded until 5 d after farrowing. Sows assigned to treatment 1 were fed one meal of 2.27 kg per day at 600 h. Sows assigned to treatment 2 were fed two equal meals of 1.13 kg (2.27 kg total) at 600 and 1400 h. Sows assigned to treatment 3 were fed two equal meals of 1.13 kg (2.27 kg total) at 600 and 1800 h. Sows assigned to treatment 4 were fed three equal meals of 0.77 kg (2.27 kg total) fed at 600, 1400, and 2200 h.

**Table 1.** Lactation diet formulation

Ingredient, %	
Corn	59.70
Soybean meal	24.40
Corn oil	1.60
DDGS	10.00
Calcium carbonate	1.53
Monocalcium phosphate 21%	0.63
Salt	0.53
L-Lysine HCl 78%	0.48
L-Threonine 99%	0.19
DL-Methionine 99%	0.10
L-Tryptophan 98.5%	0.04
L-Valine 98%	0.06
Vitamin/trace mineral premix with Phytase	0.25
Choline chloride 60%	0.10
Laxative (or Mg K SO4)	0.35
Hydrated sodium calcium aluminosilicate	0.08
Total	100.00

**Table 2.** Calculated nutrient composition of the experimental lactation diet (as-fed basis)

Composition^*a*^	
Metabolizable energy, kcal/kg	3,344
Net energy swine (NRC), kcal/kg	2,489
Moisture, %	12.81
Crude protein, %	19.54
Crude fat, %	4.42
Ash	5.77
Neutral detergent fiber (NDF), %	9.52
Analyzed calcium, %	0.80
Phosphorus, %	0.53
Analyzed Ca:P	1.50
STTD phosphorus, %	0.43
Phytase, FTU/kg	750
Zinc, ppm	157.00
Iron, ppm	209.42
Manganese, ppm	63.09
Copper, ppm	21.15
Sodium, %	0.24
Total lysine, %	1.300
Total isoleucine, %	0.758
Total leucine, %	1.704
Total methionine + cysteine, %	0.717
Total threonine, %	0.867
Total tryptophan, %	0.248
Total valine, %	0.921
SID lysine, %	1.150
SID Lys: Cal ME ratio, g/Mcal	3.541
SID amino acid: SID lysine (ratio)	
SID Met:Lys	0.32
SID Met + Cys:Lys	0.53
SID Trp:Lys	0.19
SID Thr:Lys	0.64
SID Leu:Lys	1.28
SID Ile:Lys	0.56
SID Val:Lys	0.67

^*a*^Diets were fed in meal form and manufactured at a commercial feed mill.

**Figure 1.**
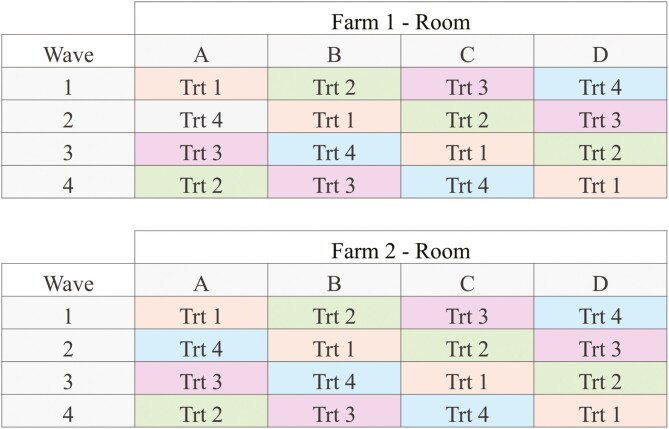
Treatments were assigned to rooms in a replicated 4 × 4 Latin Square arrangement of treatments where each treatment was fed in each room one time at each farm.

Sows were moved into farrowing rooms based on the breeding date when they reached approximately 112 d of gestation. Farrowing rooms were assigned to treatments so that each treatment was represented in each room one time during each of the four waves at each of the respective farms (Figure 1). The experiment was repeated eight times, four waves at Farm 1 and four waves at Farm 2. Because each treatment was fed in each room during one of the four waves at each farm, the experimental design was a 4 × 4 replicated Latin square (Figure 1). There were 758 sows used at farm 1 (193 T1, 190 T2,188 T3, 186 T4) and 743 sows used at farm 2 (181 T1,190 T2,181 T3, 191 T4). Overall, 374 sows were fed treatment 1, 380 sows were fed treatment 2, 370 sows were fed treatment 3, and 377 sows were fed treatment 4. All sows were fed at their respective time with the respective amount of feed using a calibrated volumetric scoop at farm 1 and from a Quattro Opti Gestal feeder (JYGA technologies, Qc, Canada) at farm 2.

### Farrowing and Data Collection

Sows were monitored every 30 min between 0600 and 1500 h to record the time the first pig was born. Sows were not provided supervision from 1501 to 0559 h. Sows corresponding with treatments that were fed between 1501 and 0559 h were only fed and not provided additional supervision at those times. Therefore, sows that farrowed overnight were checked the following morning to determine overnight farrowing events. Sows that had not yet farrowed by day 116 of gestation were induced with PGF2α (2c.c., Lutalyse, Zoetis Inc., Kalamazoo, MI). The total number of pigs born, the total number of pigs born alive, stillbirths, and mummies were recorded for each litter within 24 h of farrowing. Live pigs were weighed as a group to record litter birth weight. Adequate colostrum intake was ensured for all pigs by following the Carthage System’s standard operating procedure (SOP) for day 1 pig care. During and shortly after parturition, pigs were placed at the underline of the sow to encourage nursing. Pigs that were suspected of not nursing were placed under a heat lamp or in a warming box if chilled. A split-suckling approach was used where pigs that had been observed nursing were moved to a warming box to ensure all pigs had access to colostrum. Those that were not observed in nursing were marked and placed at the underline of the sow. No pigs were away from the sow for more than 45 min.

### Cross-Fostering Procedure

A modified management change to reduce exposure to bacteria to eliminate losses from PRRS (McREBEL) program was used at Farm 2 during waves 1, 2, and 3. No piglet movements (i.e., no cross-fostering) were permitted to equalize litter size. Falloff piglets (pigs that experienced excess weight loss or became injured) were not removed from the litter and moved to another sow in waves 1, 2, or 3 at Farm 2. Any piglet that would have been categorized as a falloff was humanely euthanized. All pigs born to a sow stayed with the birth sow for the entirety of the lactation period unless the pig was euthanized as a falloff. Starting litter inventory was calculated by subtracting the number of pigs that died prior to 48 h from the number of pigs born alive. The date, weight, and reason for mortality were recorded for all pigs until 5 d after farrowing.

Piglets from all four waves for Farm 1 and wave four for Farm 2 were required to be observed nursing the birth sow before it could be moved (i.e., cross-fostered) to a new sow. Pigs were cross-fostered within treatment to equalize litter size within 48 h of farrowing. The number of functional teats was counted for each sow and compared to the number of pigs the sow farrowed. Pigs to be cross-fostered were weighed and carried by hand from the crate of the birth sow to the crate of the recipient sow to ensure no sow was responsible for more pigs than she had available functional teats. All movements were recorded, including the date of cross-fostering and identification of the birth and recipient sows. The number of pigs was verified and recorded upon completion of cross-fostering. Starting litter inventory was calculated by adjusting for the number of pigs cross-fostered and subtracting the number of pigs that died prior to cross-fostering from the number of pigs born alive. The date, weight, and reason for mortality were recorded for all pigs until 5 d after farrowing. Pigs that were removed due to weight loss or injury were categorized as a falloff and were weighed and moved to a sow that was not enrolled in the trial.

### Statistical Analysis

Analyses were conducted using SAS (SAS Inst. Inc., Cary, NC) version 9.4. Summary statistics for farms one and two were calculated using the Means Procedure. The least squares mean for treatment effects were analyzed as 4 × 4 replicated Latin square design (Figure 1). Sow or litter was the experimental unit for all outcome variables, and the only fixed effect was treatment. The fixed effect was analyzed using the mixed procedure of SAS. The farm was the random replicate variable, farrowing room was the random column variable, and wave (i.e., block) was the random row variable. Variation in fixed effect responses due to environmental factors was controlled by including farm, farrowing room(farm), and wave(farm) in the model as random effects. A Bonferroni adjustment was used to protect against Type 1 statistical errors for simultaneous multiple comparisons. Uncertainty of the estimates of least squares means was expressed as the maximum standard error of the mean among the four treatments. Least squares mean differences for treatments were considered statistically different at *P* < 0.05. Means separation was determined using the PDIFF option in the Mixed procedure of SAS. A Bonferroni adjustment was used to protect against Type 1 statistical errors for simultaneous multiple comparisons.

The percentage of sows that farrowed under supervision (those that farrowed between the hours of 600 h and 1400 h) and the percentage of sows that farrowed under supervision without assistance was calculated using the Glimmix procedure of SAS (SAS Inst. Inc., Cary, NC). There was no evidence of over-dispersion (i.e., variances greater than those assumed by the model) in the data set for either the percentage of sows that farrowed under supervision (1.00) or for sows that farrowed under supervision and did not require assistance (0.82) using the fit statistic of Pearson’s chi-square/degrees of freedom. A binomial distribution with a logit link function was used to calculate the least squares means of the two outcome variables. The model included the fixed effect of treatment and the random variables of farm, wave(farm), and room(farm). Means separation was determined using the PDIFF option in the Glimmix procedure, and the means output was provided using the ilink option.

## RESULTS

### Summary Statistics for Farm 1

Farm 1 had an average of 3.67 parities (Table 3). Sow body weight before farrowing was 229.25 kg. Sow BCS before farrowing was 3.04, and caliper score was 8.98 caliper units. Sows were fed 4.70 d in the farrowing room prior to farrowing. The average daily feed intake (ADFI) prior to farrowing was 2.26 kg, and the total feed intake prior to farrowing was 10.65 kg. The total number of pigs born was 16.13 pigs. There were 14.58 pigs born alive, 1.22 stillbirths, and 0.34 mummies per litter. Litter weight before cross-fostering was 19.78 kg, and there were 0.91 mortalities before cross-fostering. There were 0.69 pigs cross-fostered. These extra pigs were donated by sows that started on the study but were removed from the data set. The starting litter inventory was 14.36 pigs. There were 0.87 falloff pigs per litter and 1.37 pig mortalities, resulting in 2.24 total removed pigs during the first 5 d of lactation. Sows were on trial for approximately 10.70 total days. Total days on trial included the 4.7 d before farrow and 6 d after farrow (0 being the day of farrowing and then 5 d after farrowing). Sow ADFI after farrowing increased to 6.29 kg, and the total feed intake after farrowing was 37.74 kg. Sow ADFI for the entire trial period was 4.56 kg, and the total feed intake for the trial period was 48.37 kg (Table 3).

**Table 3.** Summary statistics for sow farm 1 on sow and litter performance

Item	Observations	Mean	SD	Minimum	Maximum	CV
Parity	757	3.67	2.51	1.00	12.00	94.05
Sow body weight before farrowing, kg	758	229.25	25.74	145.58	310.66	11.23
Sow BCS before farrowing	758	3.04	0.39	2.00	4.00	12.99
Sow caliper score before farrowing	758	8.98	1.90	3.00	15.00	21.11
Days fed before farrowing, d	758	4.70	1.31	0.00	8.00	27.86
Sow ADFI before farrowing, kg	757	2.26	0.14	1.04	3.43	6.09
Sow total intake before farrowing, kg	757	10.65	3.04	2.16	21.09	28.57
Total born, *n*	758	16.13	3.99	3.00	26.00	24.76
Born alive, *n*	758	14.58	3.66	1.00	24.00	25.10
Stillbirth, *n*	758	1.22	1.37	0.00	6.00	112.80
Mummies, *n*	758	0.34	0.66	0.00	3.00	194.83
^*a*^Litter wt before cross-fostering, kg	758	19.78	4.49	2.27	31.75	22.69
Cross-fostered pigs, *n*	758	0.69	3.55	−14.00	16.00	512.25
Mortalities before cross-fostering, *n*	757	0.91	1.41	0.00	13.00	155.06
^*b*^Starting litter inventory, *n*	757	14.36	1.82	0.00	19.00	12.68
Pig falloffs per litter, *n*	758	0.87	1.64	0.00	15.00	189.01
Total pig mortality per litter, *n*	758	1.37	1.65	0.00	13.00	120.77
^*c*^Pigs removed from sow per litter, *n*	757	2.24	2.43	0.00	21.00	108.68
Total days on trial (days before and after farrowing), d	758	10.70	1.31	6.00	14.00	12.24
Sow ADFI after farrowing, kg	758	6.29	1.25	1.25	11.03	19.83
Sow total intake after farrowing, kg	758	37.74	7.49	7.48	66.21	19.84
Sow total ADFI (before farrowing and during lactation), kg	758	4.56	0.79	1.80	7.71	17.28
Total feed intake (before farrowing and during lactation), kg	758	48.37	8.09	20.14	79.82	16.71

^*a*^Only includes piglets born alive.

^*b*^Starting inventory is the set number of piglets after cross-fostering has taken place.

^*c*^Pigs removed = falloffs and mortalities combined.

### Summary Statistics for Farm 2

Farm 2 had an average of 2.96 parities (Table 4). Sow body weight was 257.83 kg before farrowing. Sow BCS before farrowing was 3.11 units, and caliper score was 10.60 caliper units. Sows were fed 3.20 d in the farrowing room prior to farrowing. ADFI prior to farrowing was 1.98 kg, and total feed intake prior to farrowing was 6.49 kg. The feed intake being under the allotted allocation of 2.27 kg could be explained by the sows being younger parties coupled with having PRRSv. The total number of pigs born was 16.39 pigs per litter. There were 14.75 pigs born alive, 1.18 stillbirths, and 0.45 mummies per litter. Litter weight before cross-fostering was 21.68 kg, and there were 0.80 mortalities before cross-fostering. There were 0.28 pigs cross-fostered. These extra pigs were donated by sows that started on the study but were removed from the data set. The starting litter inventory was 14.23 pigs. There were 0.11 pig falloffs and 2.04 pig mortalities, resulting in a total of 2.15 pigs being removed during the first 5 d of lactation. Sows were on trial for approximately 9.20 d. Total days on trial included 3.20 d before farrowing and 6 d after farrowing (0 being the day of farrowing and then 5 d after farrowing). Sow ADFI after farrowing increased to 4.36 kg, and the total feed intake after farrowing was 26.17 kg. Sow ADFI for the entire trial period was 3.57 kg, and the total feed intake for the trial period was 32.50 kg (Table 4).

**Table 4.** Summary statistics for sow farm 2 on sow and litter performance

Item	Observations	Mean	SD	Minimum	Maximum	CV
Parity	743	2.96	1.67	1.00	10.00	85.33
Sow body weight before farrowing, kg	742	257.83	39.20	145.13	365.99	15.20
Sow BCS before farrowing	743	3.11	0.42	2.00	10.00	13.64
Sow caliper score before farrowing	743	10.60	2.15	4.00	16.00	20.32
Days fed before farrowing, d	743	3.20	1.46	0.00	8.00	45.69
Sow ADFI before farrowing, kg	726	1.98	0.44	0.00	3.78	22.12
Sow total intake before farrowing, kg	726	6.49	3.11	0.00	16.53	47.98
Total born, *n*	743	16.39	3.53	5.00	27.00	21.53
Born alive, *n*	743	14.75	3.11	4.00	25.00	21.07
Stillbirth, *n*	743	1.18	1.39	0.00	6.00	117.23
Mummies, *n*	743	0.45	0.80	0.00	4.00	175.87
^*a*^Litter wt before cross-fostering, kg	741	21.68	4.42	6.62	35.06	20.40
Cross-fostered pigs, *n*	741	0.28	1.71	−10.00	12.00	609.16
Mortalities before cross-fostering, *n*	743	0.80	1.11	0.00	6.00	138.23
^*b*^Starting litter inventory, *n*	741	14.23	2.44	3.00	22.00	17.15
Pig falloffs per litter, *n*	743	0.11	0.44	0.00	3.00	408.27
Total pig mortality per litter, *n*	743	2.04	1.98	0.00	12.00	97.11
^*c*^Pigs removed from sow per litter, *n*	743	2.15	2.01	0.00	12.00	93.39
Total days on trial (days before and after farrowing), d	743	9.20	1.46	6.00	14.00	15.90
Sow ADFI after farrowing, kg	742	4.36	1.01	0.00	3.79	23.13
Sow total intake after farrowing, kg	743	26.17	6.06	0.00	40.74	23.17
Sow total ADFI (before farrowing and during lactation), kg	743	3.57	0.78	0.76	5.55	21.81
Total feed intake (before farrowing and during lactation), kg	743	32.50	7.40	6.80	49.47	22.75

^*a*^Only includes piglets born alive.

^*b*^Starting inventory is the set number of piglets after cross-fostering has taken place.

^*c*^Pigs removed = falloffs and mortalities combined.

### Sow Measurements and Feed Consumption before Farrowing

Parity was not different (*P* = 0.81) among treatments (Table 5). Sow body weight before farrowing for treatment 3 was 5.14 kg and 4.48 kg greater (*P* ≤ 0.05) compared to treatments 1 and 2, respectively, but not different (*P* = 0.94) compared to treatment 4. Treatment 4 sow body weight before farrowing was 4.96 kg greater than treatment 1 (*P* = 0.03), but not different than treatment 2 (*P* = 0.06). Treatments 1 and 2 sow body weight before farrowing was not different (*P* = 0.77). Sow BCS before farrowing was not different among treatments (*P* = 0.06). Caliper score before farrowing was 0.39 units greater (*P* = 0.04) in treatment 3 compared to treatment 1 but not different (*P* ≥ 0.12) compared to treatments 2 and 4 (Table 5).

**Table 5.** Effects of meal frequency (treatment) prior to farrowing on sow and litter performance

	^*a*^Treatment					
Item	1×	2×	2×	3×	SEM	*P*-value
	(600 h)	(600 h & 1400 h)	(600 h & 1800 h)	(every 8 h)		
Litters, *n*	374	380	370	377		
Parity	3.24	3.37	3.29	3.36	0.11	0.81
Sow body weight before farrowing, kg	240.86^a^	241.52^ab^	246.00^c^	245.82^bc^	1.63	0.03
Sow BCS before farrowing	3.04	3.09	3.11	3.05	0.02	0.06
Sow caliper score before farrowing	9.64^ a^	9.70^ ab^	10.04^b^	9.78^ab^	0.10	0.04
Days fed before farrowing, d	4.41^b^	3.89^a^	3.67^a^	3.89^a^	0.06	<0.0001
Sow ADFI before farrowing, kg	2.02^a^	2.21^c^	2.11^b^	2.15^b^	0.02	<0.0001
Sow total intake before farrowing, kg	9.09^b^	8.62^b^	7.93^a^	8.63^b^	0.14	<0.0001
Total born, *n*	16.17	16.17	16.28	16.39	0.20	0.83
Born alive, *n*	14.6	14.57	14.59	14.87	0.18	0.59
Stillbirth, *n*	1.15	1.20	1.30	1.14	0.07	0.33
Mummies, *n*	0.42	0.40	0.39	0.38	0.04	0.89
^*b*^Litter wt before cross-fostering, kg	20.82	20.69	20.52	20.85	0.23	0.74
Cross-fostered pigs, *n*	0.41	0.56	0.65	0.37	0.14	0.48
Mortalities before cross-fostering, *n*	0.87	0.82	0.89	0.85	0.07	0.87
^*c*^Starting litter inventory, *n*	14.14	14.31	14.35	14.38	0.11	0.42
Pig falloffs per litter, *n*	0.43^a^	0.69^b^	0.37^a^	0.47^ab^	0.06	<0.01
Total pig mortality per litter, *n*	1.74	1.67	1.67	1.73	0.09	0.93
^*d*^Pigs removed from sow per litter, *n*	2.17	2.36	2.04	2.21	0.12	0.26
Total days on trial (days before and after farrowing), d	10.41^a^	9.86^b^	9.67^b^	9.89^b^	0.06	<0.0001
Sow ADFI after farrowing, kg	5.36	5.32	5.29	5.35	0.06	0.76
Sow total intake after farrowing, kg	32.18	31.92	31.71	32.1	0.33	0.76
Sow total ADFI (before farrowing and during lactation), kg	3.97	4.10	4.10	4.09	0.04	0.05
Total feed intake (before farrowing and during lactation), kg	41.26^b^	40.49^ab^	39.56^a^	40.60^ab^	0.38	0.02

Least squares means within a row that do not share a common superscript differ (*P* < 0.05).

^*a*^All sows were allocated 2.27 kg of complete feed per day prior to farrowing. Treatment was the number of meals provided throughout the day. 2.27 kg were divided into equal-sized meals based on treatment assignment.

^*b*^Only includes piglets born alive.

^*c*^Starting inventory is the set number of piglets after cross-fostering has taken place.

^*d*^Pigs removed = falloffs and mortalities combined.

Sows assigned to treatment 1 were fed at least 0.52 d longer (*P* < 0.0001) compared to all other treatments (Table 5). The number of days fed before farrowing was not different (*P* ≥ 0.08) among treatments 2, 3, or 4. ADFI before farrowing for treatment 2 was at least 0.06 kg greater (*P *≤ 0.001) compared to all other treatments. ADFI before farrowing of sows assigned to treatment 4 was 0.13 kg greater (*P* ≤ 0.05) compared to sows assigned to treatment 1 but not different (*P* = 0.68) compared to sows assigned to treatment 3. Treatment 3 ADFI before farrowing was 0.09 kg greater (*P* < 0.001) than treatment 1. Total feed intake before farrowing was not different (*P* ≥ 0.14) among treatments 1, 2, and 4, but all were at least 0.69 kg greater (*P* < 0.01) than treatment 3 (Table 5).

### Farrowing Performance

**Figure 2.**
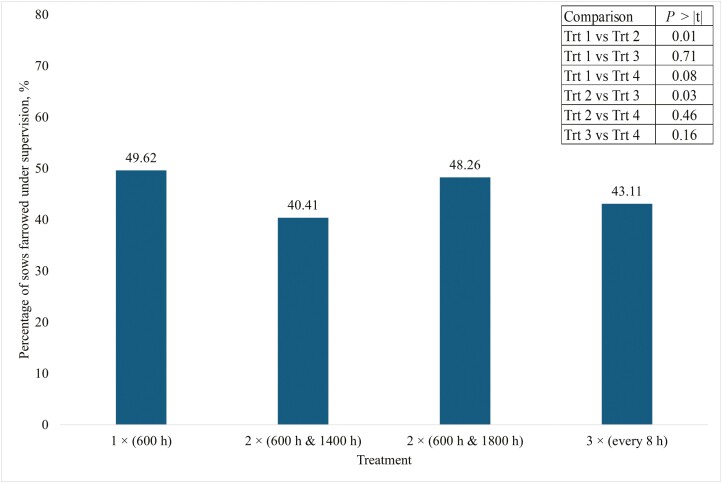
Percentage of sows that farrowed under supervision during the day across all treatments. Treatment 1 sows were fed one meal of 2.27 kg per day at 600 h. Treatment 2 sows were fed two equal amounts of 1.13 kg (2.27 kg total) at 600 and 1400 h. Treatment 3 sows were fed two equal meals of 1.13 kg (2.27 kg total) at 600 and 1800 h. Treatment 4 sows were fed three equal meals of 0.77 (2.27 kg total) fed at 600, 1400, and 2200 h.

The total number of pigs born per litter and the number of pigs born alive were not different (*P* ≥ 0.59) among treatments (Table 5). The number of stillborn pigs was not different (*P* = 0.33) among treatment 1 (1.15 pigs), treatment 2 (1.20 pigs), treatment 3 (1.30 pigs), and treatment 4 (1.14 pigs). The number of mummies did not differ (*P* = 0.89) among treatments. Litter weight prior to cross-fostering was not different (*P* = 0.74) among treatments, and mortalities prior to cross-fostering were also not different (*P* = 0.87) among treatments. The number of pigs cross-fostered was not different among treatments (*P* = 0.48). Less than half of all sows in the trial farrowed under supervision (Figure 2). However, the percentage of sows that farrowed under supervision differed among treatments (Figure 2). The mean comparisons (least significant difference) of the estimated probabilities of sows that farrowed under supervision for sows assigned to treatment 1 and treatment 3 was greater (*P* ≤ 0.03) than the percentage of sows assigned to treatment 2. There were no differences in the percentage of sows that farrowed under supervision between treatments 1 and 3 (*P* = 0.71), 2 and 4 (*P* = 0.46), or between 3 and 4 (*P* = 0.16, Figure 2). Feeding sows 3 times per day at 8 h intervals (treatment 4) tended (*P* = 0.08) to reduce the percentage of sows farrowed under supervision compared with sows fed once a day (treatment 1).

**Figure 3.**
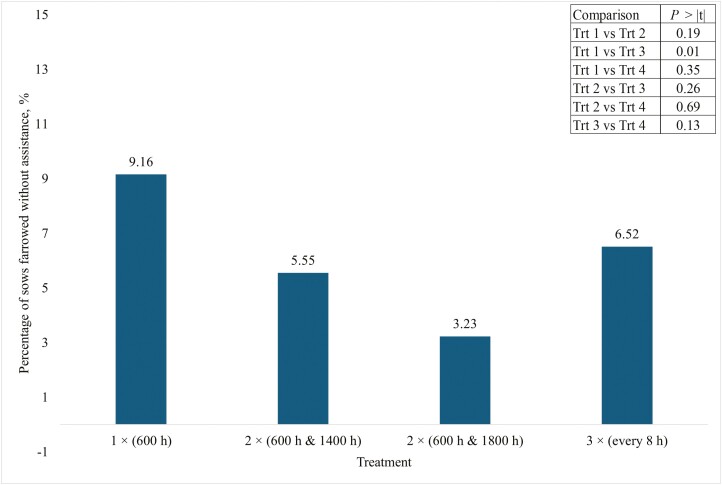
Percentage of sows that farrowed under supervision and without assistance across all treatments. Treatment 1 sows were fed one meal of 2.27 kg per day at 600 h. Treatment 2 sows were fed two equal amounts of 1.13 kg (2.27 kg total) at 600 and 1400 h. Treatment 3 sows were fed two equal meals of 1.13 kg (2.27 kg total) at 600 and 1800 h. Treatment 4 sows were fed three equal meals of 0.77 (2.27 kg total) fed at 600, 1400, and 2200 h.

Over 90% of sows that farrowed under supervision were provided assistance (Figure 3). There were no differences in the percentage of sows provided assistance between treatments 1 and 2 (*P* = 0.19), treatments 1 and 4 (*P* = 0.35), treatments 2 and 3 (*P* = 0.26), treatments 2 and 4 (*P* = 0.69) or treatments 3 and 4 (*P* = 0.13, Figure 3). Feeding sows twice per day at 12 h intervals (treatment 3) reduced (*P* = 0.01) the percentage of sows provided assistance compared with feeding sows once per day (treatment 1, Figure 3).

### Litter Performance and Sow Feed Consumption after Farrowing

Starting litter inventory after cross-fostering was not different (*P* = 0.42) among treatments (Table 5). The number of fall-off pigs for treatment 2 was 0.26 pigs and 0.32 pigs more (*P* ≤ 0.02) than for treatments 1 and 3, respectively. Total pig mortality per litter was not different (*P *= 0.93) among treatments. The number of pigs removed from each sow per litter was not different (*P* = 0.26) among treatments. Total days on trial were longer (*P* < 0.0001) by at least 0.53 d for treatment 1 compared to all other treatments. Total days on trial did not differ (*P *≥ 0.08) among treatments 2, 3, and 4. Sow total ADFI and total feed intake after farrowing was not different (*P* = 0.76) among treatments. Sow ADFI from the time the sows were moved into the farrowing room until 5 d after farrowing was not different among treatments (*P* = 0.05). Sow total feed intake from the time the sows were moved into the farrowing room until 5 d after farrowing was 1.7 kg greater (*P* = 0.01) in treatment 1 compared to treatment 3, but not different (*P* ≥ 0.87) compared to treatments 2 and 4. Sow total intake for treatments 2, 3, and 4 did not differ (*P* ≥ 0.30, Table 5).

## DISCUSSION

This study aimed to determine if stillborn pigs per litter would decrease as the interval between a sow’s last meal and farrowing also decreased, even when overall feed allocation was maintained. Feeding strategies in late gestation to aid sows through a successful farrowing event by providing them with the requisite energy has been extensively researched (Gourley et al., 2020; Miller and Kellner, 2020; Oliveria et al., 2020; Tucker et al., 2022). However, there is no consensus among published literature on the effectiveness of increasing meal frequency prior to farrowing in reducing stillbirths. Increasing the frequency of feeding can be more labor-intensive for a farm that feeds sows from a feed cart or some other manual delivery system. Additionally, these farms are likely limited to only feeding during staffed hours. Therefore, it was important to understand if the extra time and money invested in labor to increase feed frequency resulted in more piglets being born alive and the opportunity for a greater number of pigs to be weaned.

This study replicated the trial at two farms with different PRRSv statuses. Differences in feed intake and body weights between farms may be related to PRRSv status. Although not statistically compared, farm 1 sows had greater feed intakes during the early stages of lactation compared to farm 2. Sows from farm 2 were heavier at the time of farrowing compared with sows from farm 1. This is also likely an artifact of the PRRSv infection. The introduction of new sows from outside of farm 2 for replacement was prohibited during the PRRSv elimination program. Replacement gilts were raised and gestated in a gilt development unit (GDU). Gilts in the GDU were provided ad libitum access to feed during gestation, which resulted in a heavier body weight at farrowing than a typical first parity sow. The increase in body weight into farrowing resulted in a greater than ideal BCS for first parity sows at farm 2. Sows that have ad libitum access to feed during gestation are expected to be fatter entering farrowing compared with sows that are limit-fed during gestation. Fatter sows have lesser voluntary feed intakes compared with thinner sows during lactation (Eissen et al., 2020). Differences in lactation feed intake were not statistically compared, yet sows at farm 2 were heavier and fatter, and they consumed less feed during the early stages of lactation. These differences could be related to the PRRSv infection causing changes to management practices at farm 2 compared with farm 1 or could simply be an anomaly in this group of sows. True differences cannot be determined from these data.

Farrowing performances, including total pigs born, pigs born alive, the number of stillbirths, and mummies, were numerically similar between the farms, although performances were not statistically compared.

Sows assigned to treatment 1 were fed the treatment allocation prior to farrowing at least 0.52 d longer than the other 3 treatment groups (Table 5). This difference in the duration of the treatment period is a result of the research protocol. Sows on treatment 1 were provided with their full daily allocation of feed at 600 h. If that sow was fed at 600 h she was counted as not farrowing on that respective day. On the other hand, if sows on treatments 2, 3, and 4 began farrowing prior to the full allocation of their daily meal allotment, they were immediately provided ad libitum access to feed, and the prior day was used to calculate the duration of their prefarrow treatments. This resulted in a mathematical reduction in the ADFI prior to the farrowing of sows on treatment 1 compared with sows on the other three treatments.

Gourley et al. (2020) reported no difference in total feed consumed prior to farrowing for sows fed once at 0700 h or sows fed four times at 0100, 0700, 1300, and 1900 h. The discrepancy could be due to experimental methodologies. This study considered the farrow date as the day the last pig was observed after farrowing was complete. Sows that started farrowing in the night would have a farrow date the next morning when the full litter was observed. This is potentially a reason for the differences in feed intake for treatments 2 to 4, as the sow may not have received all allocated meals before the start of farrowing. When this occurred, sows were immediately transitioned to ad libitum access to feed. Feed provided prior to the start of farrowing was included in the total feed consumed before farrowing. All feed provided after the sow was transitioned to ad libitum access to feed was included in the total feed consumed after farrowing. This reduced the number of days fed prior to farrowing for sows assigned to treatments 2, 3, and 4 compared with sows assigned to treatment 1. Whereas if a sow on treatment 1 started farrowing after she was fed at 600 h, the entire 2.27 kg feed allocation was counted toward the total feed consumed before farrowing and that day was counted towards days fed before farrowing.

The number of pigs stillborn was not different among treatments in this study. Gourley et al. (2020) used 3 different feeding strategies being fed one delivery of 2.7 kg, four deliveries of 0.68 kg (2.7 kg total), and four deliveries of ad libitum access to feed (approximately 3.34 kg ADFI before farrowing). Gourley et al. (2020) ultimately reduced the interval between the last meal and farrow from 605 min to 196 min and had no difference in stillborn rate, which is in agreement with the current study. Conversely, Feyera et al. (2018) reported an increased probability of stillbirth as the interval from the last meal to farrowing doubled from 4% at less than 3 h since the last meal to 8% over 6 h since the last meal. Tucker et al. (2022) reported that sows fed twice a day with shorter farrowing durations had fewer stillbirths compared to those fed once a day with shorter farrowing durations. However, stillborn numbers were not different when sows with both short and long farrowing durations (LFD) were combined regardless of feeding frequency. Therefore, farrowing duration was most likely driving the differences in the number of stillbirths rather than feeding frequency.

Only reducing the interval from the last meal to farrowing was not enough to decrease stillbirths. Gourley et al. (2020) provided a diet with similar SID Lys (1.15%) and ME (3320 kcal/kg) to the current study. However, Gourley et al. (2020) had sows that were fed at least 16% more feed than the current study. Tucker et al. (2022) provided 1.01% SID Lys and 3568 kcal/kg DE, and the sows were fed 60% more feed than the current study. Miller and Kellner (2020) changed total feed allocations along with feeding frequency from the lowest 1.8 kg/d to 5.4 kg/d. Changing total feed allocations along with feeding frequency potentially confounded the results of feeding frequency. Nutrient density and volume of feed need to also be considered in the transition period.

Though not recorded in the present study, prior work has reported a link between farrowing duration, birth interval, and stillbirth rate. It is estimated that 88% of piglets identified as stillborn die due to intrapartum asphyxiation during active farrowing (Rangstrup-Christensen et al., 2018). Ju et al. (2021) reported that sows with a total born <5 or >22 had the longest farrowing durations, and farrowing durations that exceeded 4 h had an increase in stillbirths. Liu et al. (2021) reported that sows with short farrowing durations (SFD;<300 min) had significantly reduced time from the last meal until the onset of farrowing compared to the LFD ( >300 min) sows. These sows were fed twice a day, and they had a total of 3.0 kg per day compared to the current study, where sows were fed 2.27 kg per day at their corresponding times. Liu et al. (2021) reported these SFD sows had higher glucose concentrations at farrowing, whereas LFD sows had partially depleted glucose concentrations at the onset of farrowing. The SFD sows obtained more energy from the blood and used a greater proportion of glucogenic energy and less ketogenic energy, which most likely allowed them to farrow faster. Additionally, Liu et al. (2021) reported the higher concentration of plasma lactic acid at farrowing in LFD sows most likely reflected that insufficient glucose (energy) was available to fuel the uterine contractions, which then caused the piglets to be captured in the birth canal and possibly die from intrapartum asphyxiation. Accordingly, the lower plasma lactic acid concentration at farrowing in SFD sows reflected a sufficient supply of glucogenic energy, which in turn allowed stronger uterine contractility and piglets to be expelled efficiently through the birth canal.

If the total number of births continues to rise, the birth interval needs to be reduced to maintain a farrowing duration under 4 h. However, the birth interval is ultimately dependent on the fluctuation of hormones such as oxytocin (Algers and Uvnäs-Moberg, 2007). The binding of oxytocin to its receptors leads to activation of the inositol triphosphate pathway. Inositol triphosphate triggers ionized calcium (Ca2þ) release from intracellular storage with the formation of Ca2þ-calmodulin complexes, which in turn trigger activation of myosin light-chain kinase activity that initiates smooth muscle contraction (Gimpl and Fahrenholz, 2001). Changes to the oxytocin cascade that initiates smooth muscle contract is unlikely to change. Thus, farrowing duration is likely to increase with increasing litter size.

A large percentage of preweaning mortality occurs within the first 3 d of farrowing (Koketsu et al., 2006; Shankar et al., 2009). This trial followed sows for the first 5 d to account for mortalities that were more likely directly related to farrowing. After that, mortalities become more related to being laid on or lack of access to the underline (Muns et al., 2016). In this study treatment 2 had a greater number of fall-off pigs but also the least mortalities, ultimately making total pig removal per sow not different among all treatments. Gourley et al. (2020) reported no differences in mortalities within 24 h after birth but reported a reduction in prewean mortalities for sows that were fed multiple times a day compared to once a day from 24 h after birth to wean. It is likely the difference is due to other factors not related to the farrowing or the frequency of feedings before farrowing. Feeding sows once a day in this trial resulted in a greater caloric intake, but that did not aid in piglet survivability. Therefore, meal frequency before farrow is likely not related to pig survivability.

Less than 50% of all sows in the current trial farrowed under supervision (between 0600 and 1500 h) across all treatments (Figure 2). Although there were differences in the percentage of sows that farrowed under supervision and without assistance among treatments (Figure 3), decreasing the interval between the last meal and farrowing by increasing meal frequency did not consistently result in an increase in the number of sows that farrowed without assistance. Carthage’s SOP for assisting is if the birthing interval exceeds 20 to 25 min for a normal sow and 15 min for a high-risk sow (above parity six, or three or more stillbirths in past farrowing events) during active farrowing. These sows had an average of 16 total pigs. To maintain a 4 h farrowing duration, a pig should be born every 15 min. The 20 to 25 timeframe before providing assistance gives the sow enough time to have a pig on her own if she is able but requires intervention before the welfare of the pigs becomes compromised. Tucker et al. (2022) only provided assistance after a sow showed distress or 45 min had passed since the last piglet was born. Gourley et al. (2020) assisted after 30 to 45 min of no farrowing progress. It is possible that farm management practices are confounding these results.

## CONCLUSION

Reducing the interval between a sow’s last meal and farrowing by increasing the frequency of feeding in very late gestation did not reduce the number of stillborn pigs. The inconsistency in published literature suggests that the stillbirth rate is a complex multifactorial trait influenced by many physiologic and management factors. Investing in farm labor to feed sows multiple times per day may not be justified based on the findings of this trial. It is important to note that animal husbandry practices must be maintained to ensure sow and piglet welfare, even when feeding sows once per day.
